# Requirement for *Dlgh-1* in Planar Cell Polarity and Skeletogenesis during Vertebrate Development

**DOI:** 10.1371/journal.pone.0054410

**Published:** 2013-01-22

**Authors:** Charlene Rivera, Sara J. S. Simonson, Idella F. Yamben, Shalini Shatadal, Minh M. Nguyen, Maryline Beurg, Paul F. Lambert, Anne E. Griep

**Affiliations:** 1 Department of Cell and Regenerative Biology, University of Wisconsin-Madison, Madison, Wisconsin, United States of America; 2 Department of Oncology, University of Wisconsin-Madison, Madison, Wisconsin, United States of America; 3 Institut National de la Santé et de la Recherche Médicale, Hôpital Pellegrin, Bordeaux, France; Indiana University School of Medicine, United States of America

## Abstract

The development of specialized organs is tightly linked to the regulation of cell growth, orientation, migration and adhesion during embryogenesis. In addition, the directed movements of cells and their orientation within the plane of a tissue, termed planar cell polarity (PCP), appear to be crucial for the proper formation of the body plan. In *Drosophila* embryogenesis, *Discs large* (*dlg*) plays a critical role in apical-basal cell polarity, cell adhesion and cell proliferation. Craniofacial defects in mice carrying an insertional mutation in *Dlgh-1* suggest that Dlgh-1 is required for vertebrate development. To determine what roles Dlgh-1 plays in vertebrate development, we generated mice carrying a null mutation in *Dlgh-1*. We found that deletion of *Dlgh-1* caused open eyelids, open neural tube, and misorientation of cochlear hair cell stereociliary bundles, indicative of defects in planar cell polarity (PCP). Deletion of *Dlgh-1* also caused skeletal defects throughout the embryo. These findings identify novel roles for *Dlgh-1* in vertebrates that differ from its well-characterized roles in invertebrates and suggest that the *Dlgh-1* null mouse may be a useful animal model to study certain human congenital birth defects.

## Introduction

The development of specialized organs in vertebrates is tightly linked to the regulation of cell growth, apical-basal cell polarity and cell-cell adhesion during embryogenesis. In addition, the directed movements of cells and their orientation in the same direction within the plane of a tissue, termed planar cell polarity (PCP), appear to be crucial for the proper formation of the body plan. Of great interest has been to decipher the mechanisms involved in regulating these critical aspects of development. From studies in invertebrates it is known that certain **P**SD95/**D**lg/**Z**O-1 (PDZ) domain containing proteins such as Discs-large (Dlg) play prominent roles in regulating apical-basal polarity [1], [2] while different PDZ proteins are part of a genetic network that regulates PCP [3]. Recent work in vertebrates supports the contention that the function of many polarity factors has been conserved cross-species. However, studies also suggest that certain factors have different or additional roles in vertebrates as compared to invertebrates [4]. In this study, we address the role of *Dlgh-1*, the mouse homolog of *Drosophila dlg*, in mouse development.

In *Drosophila melanogaster*, mutations in *dlg* and *scrib*, the gene encoding a second PDZ protein, Scribble, result in neoplastic overgrowth, multilayering and loss of cell shape in various epithelial tissues including the imaginal discs [1], the embryonic epidermis, and the follicular epithelia [2], suggesting a role for these factors in cell proliferation, adhesion and apico-basal polarity. In *Drosophila* and *Caenorhabditis elegans*, Dlg and Scrib are thought to be required for properly localizing and maintaining adherens junctions and apical determinants [1], [2], [5], [6], [7], [8], [9]. In *Drosophila*, Dlg localizes to the septate junctions where it forms a complex with Scrib and Lethal giant larvae (Lgl) [1], [2]. This Dlg/Scrib/Lgl complex antagonizes other PDZ complexes, thereby restricting adherens junctions and apical determinants, such as Crumbs, to the appropriate compartment [10]. Finally, the Partner of Inscuteable/Discs-large complex is required for planar polarity during the asymmetric division of the sensory organ precursor (pl) cell [11].

Dlg is a member of the membrane-associated guanylate kinase (MAGUK) family [12], and, as a scaffolding protein, is capable of assembling supramolecular complexes at specific sites within and proximal to the cytoplasmic membrane. Dlg may accomplish this by interacting with other molecules through its multiple protein-protein interaction domains, which include three PDZ domains, a src-homology 3 (SH3) domain, a band 4.1 domain, and a guanylate kinase homolog (GUK) domain [12]. Homologs of *Drosophila* Dlg are found in humans (hDlg), rat (SAP97), and mouse (Dlgh-1, also referred to as Dlg-1), and the high degree of conservation among its multiple domains suggests conserved function cross-species [13], [14], [15]. In mammals, Dlgh-1 is ubiquitously expressed and is found at sites of cell-cell contacts in epithelial and neuronal cells [16], [17], [18], [19], [20], [21]. It interacts with a wide variety of proteins, which include the tumor suppressors adenomatous poli (APC) [22] and protein tyrosine phosphatase and tensin homologue (PTEN) [23], Shaker-type K+ channel α-subunits [24], the G-protein coupled receptor homologue tumor endothelial marker 5 (TEM5) [25], and the MAGUK Lin-2/CASK [26]. In addition to interacting with endogenous cellular proteins, Dlgh-1 is also targeted by multiple human viral oncoproteins including the human papillomavirus (HPV) E6 [27], the adenovirus E4-ORF1 [28], and the human T cell leukemia virus type 1 (HTLV-1), Tax 1, [29]. These interactions have been implicated in the transforming and tumorigenic properties of the viral oncoproteins [27], [29], [30], [31], [32], [33].

The role of *Dlgh-1* in mouse development has begun to be explored. Caruana and Bernstein [34] reported that mice carrying a gene trap insertion in *Dlgh-1* (*Dlgh-1^g/gtt^* mice), which results in a Dlgh-1 protein lacking the SH3, band 4.1, and GUK domains, exhibited a cleft palate and a shorter mandible than *Dlgh-1* wild type mice. Additional studies on this *Dlgh-1^gt/gt^* mouse showed that these mice also exhibited a decrease in the number of nephrons in the developing kidney [35] and cell cycle misregulation in the epithelium of the ocular lens [36]. Since the L27 and three PDZ domains of Dlgh-1 were left intact in the gene trap allele [34], [37], it is possible that the fusion protein generated from this gene trap allele may retain some or acquire new activities of Dlgh-1 and, therefore, the phenotypes observed may not fully reflect the null phenotype. Consistent with this possibility, lacZ staining of *Dlgh-1^gt/gt^* lenses and control *Dlgh-1^+/+^* lenses showed similar staining patterns (Rivera and Griep, unpublished observations). Furthermore, two recent reports show that deletion of *Dlgh-1* in mice caused abnormalities in the ureteric smooth muscle [38] and absence of vagina and seminal vesicles [39]. Additionally, conditional deletion of *Dlgh-1* in the lens led to more severe phenotype than was observed in the lenses of the *Dlgh-1^gt/gt^* mice including epithelial and fiber cell specific defects in cell adhesion and polarity [40].

In the present study, we generated mice carrying a germline null mutation in *Dlgh-1* by deleting one of the exons encoding the first PDZ domain and characterized the wide-ranging effects of this mutation on mouse embryogenesis. Loss of *Dlgh-1* not only recapitulated the craniofacial and kidney defects observed in the *Dlgh-1^gt/gt^* mouse but also resulted in reduced ossification in the skull, maxilla, and middle ear, and shortening of the long bones. Furthermore, deletion of *Dlgh-1* resulted in eyelid closure and neural tube closure defects, and the misorientation of the stereociliary bundles of the cochlear hair cells, all of which suggest defects in PCP. Thus, our analysis identifies novel roles for *Dlgh-1* in mouse development, places Dlgh-1 in the group of factors that regulate PCP in the mouse, and provides new insight into the distinct *in vivo* requirements for this gene in vertebrates, as compared to invertebrates.

## Results

### Generation of *Dlgh-1* Null Mice

**Figure 1:**
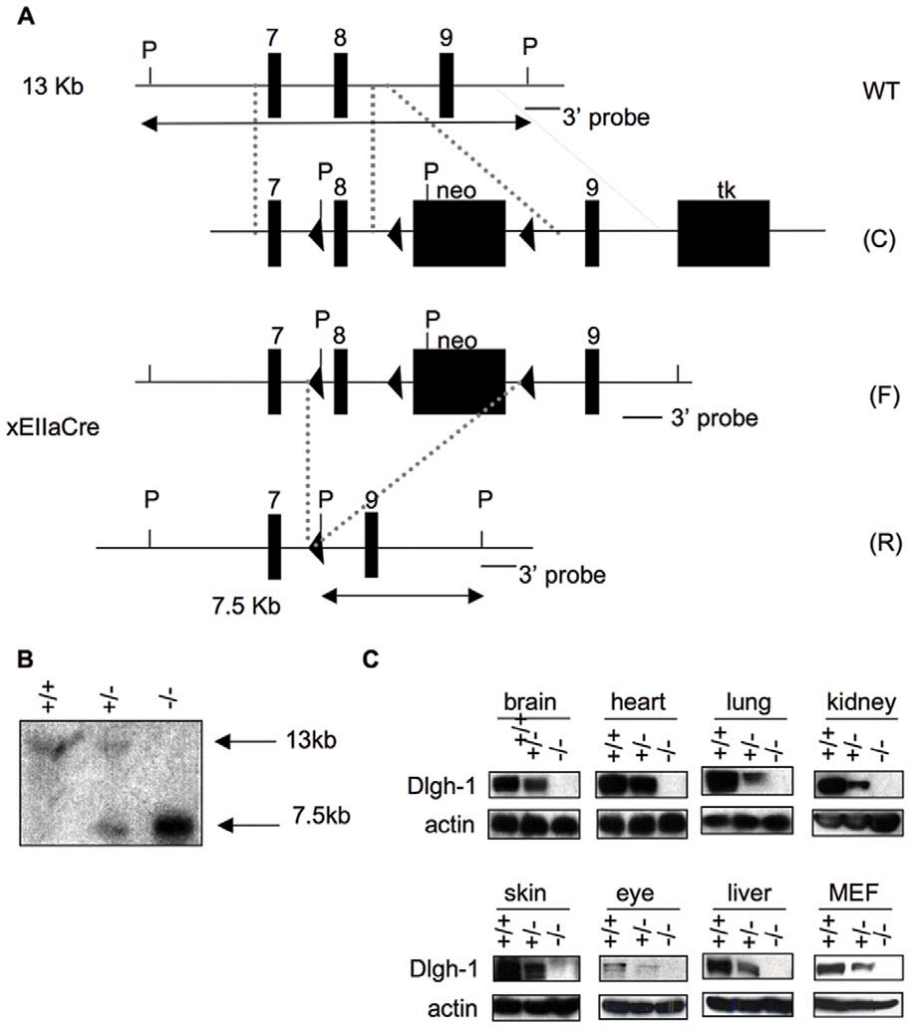
Generation of *Dlgh-1* null mice. (A) Schematic representation of the targeting of the *Dlgh-1* allele. WT, wild-type allele; C, construct, plasmid containing loxP sites flanking exon 8 of *Dlgh-1* and neo cassette, and the TK cassette; F, the floxed allele allele, containing lox P sites flanking *Dlgh-1′*s exon 8 and the neo cassette; R, recombined allele, desired recombination event lacking exon 8 and neo. Abbreviations: 7, exon 7; 8, exon 8; 9, exon 9; P, *Pst*I; triangles, lox P sequences; neo, neomycin; TK, thymidine kinase. 3′ probe for Southern blot analysis is denoted by black bars. Fragments expected from *Pst*I digestion and 3′ probe hybridization are depicted by double-arrowed lines. (B) Southern blot analysis of *Pst*I digested genomic DNA isolated from mice that were WT (+/+), heterozygote (+/−), or null (−/−) for the *Dlgh-1* allele. Sizes of hybridizing bands are shown in kb. (C) Western blot analysis of Dlgh-1 protein levels. Protein lysates of brain, heart, lung, kidney, skin, eye, liver, and mouse embryo fibroblasts (MEF) from *Dlgh-1* wild type (+/+), heterozygote (+/−), or null (−/−) mice were resolved by SDS-PAGE and immunoblotted with an anti-Dlgh-1 antibody, as described in Materials and Methods. Membranes were reprobed with an anti-β-actin antibody as a loading control.

To generate *Dlgh-1* null mice, mice carrying a mutant allele of *Dlgh-1*, in which exon 8 and a neomycin resistance-cassette are flanked by loxP sites [40] (Fig. 1A), were mated to *EIIA-Cre* transgenic mice [41]. The *Dlgh-1* null allele was moved onto the FVB/NJ and C57BL/6J genetic backgrounds by backcrossing to either FVB/NJ or C57BL/6J mice for at least three generations before use. Mice heterozygous for the null allele were intercrossed to generate *Dlgh-1^−/−^* mice and Southern blot analysis was used to confirm the genotype of the progeny (Fig. 1B).

To determine if the mutated *Dlgh-1* allele was indeed a null allele, Dlg-1 protein levels in brain, heart, lung, kidney, skin, liver, and eyes of E18.5 *Dlgh-1^+/+^*, *Dlgh-1^+/−^*, and *Dlgh-1^−/−^* mice were assessed by immunoblot analysis using an N-terminal specific anti-Dlgh-1 antibody. The Dlgh-1 protein levels were reduced by approximately 50% in the *Dlgh-1^+/−^* mice compared to the *Dlgh-1^+/+^* mice and Dlgh-1 protein was not detected in any of the tissues analyzed from the *Dlgh-1^−/−^* mice or in mouse embryo fibroblasts (MEFs) derived from day E14.5 *Dlgh-1* null embryos (Fig. 1C). These results confirmed that the *Dlgh-1* mutant allele is a null allele.

### Effect of Loss of *Dlgh-1* on Mouse Embryogenesis

It has been reported that mice homozygous for the *Dlgh-1^gt^* allele die at or near the time of birth [12]. To determine if nullizygosity for *Dlgh-1* also would be lethal, we intercrossed *Dlgh-1^+/−^* mice and examined litters for live *Dlgh-1^−/−^* pups. Of the 47 pups genotyped, 10 were *Dlgh-1^+/+^* (21%), 22 (47%) were *Dlgh-1^+/−^*, and 15 (32%) were *Dlgh-1^−/−^*. Of the 15 *Dlgh-1^−/−^* pups, all of which exhibited severe craniofacial abnormalities, 13 were found dead on the day of birth and the remaining two died shortly after being found. These data indicate that loss of *Dlgh-1* confers perinatal lethality with 100% penetrance and support the hypothesis that *Dlgh-1* plays an important role in mouse development.

**Figure 2:**
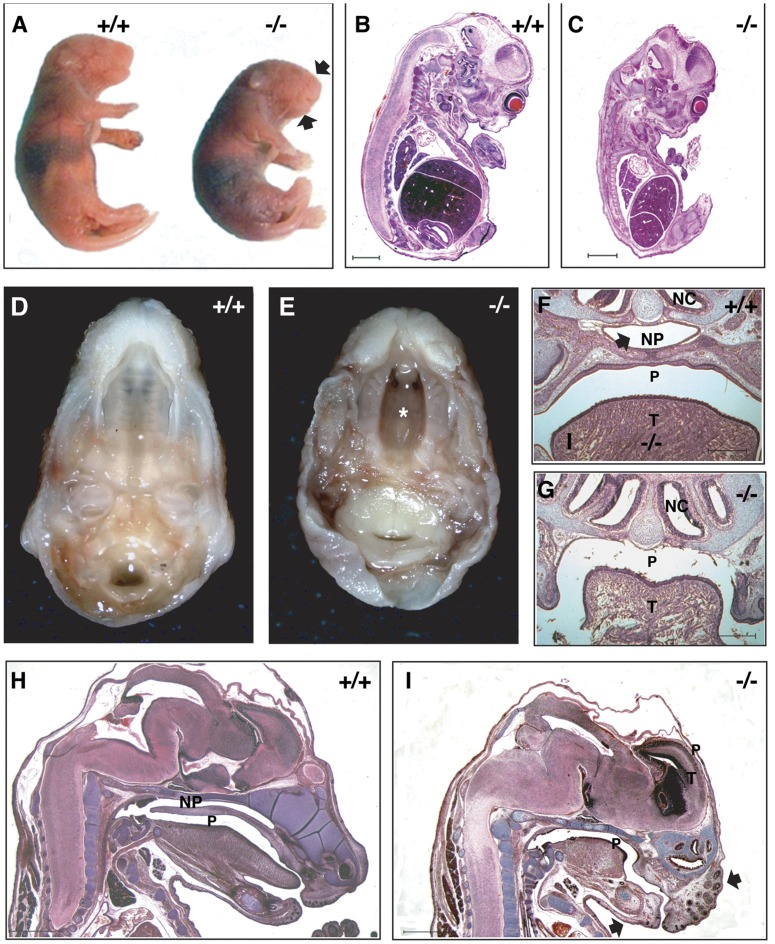
Phenotype of *Dlgh-1^−/−^* mice. (A) Newborn *Dlgh-1^−/−^* mice are smaller then their *Dlgh-1^+/+^* counterparts and have a shortened snout and mandible (arrows). (B–C) Hematoxylin and eosin stained paraffin sections of FVB/NJ E14.5 *Dlgh-1^+/+^* (B) and *Dlgh-1^−/−^* (C) mice. At this developmental stage, the reduced size of the *Dlgh-1^−/−^* is noticeable. (D–E). Dissected heads of *Dlgh-1^+/+^* and *Dlgh-1^−/−^* C57BL/6J E18.5 embryos showing the cleft palate (asterisk*) in *Dlgh-1^−/−^* mice. (F–G) Frontal sections of *Dlgh-1^+/+^* (F) and *Dlgh-1^−/−^* (G) E18.5 embryos on the C57BL/6J background showing the absence of the nasopharyngeal passage (NP, arrow in F). (H–I) Sagittal H&E stained sections of E18.5 *Dlgh-1^+/+^* (H) and *Dlgh-1^−/−^* (I) embryos on the FVB/NJ background showing the dome-shaped head of *Dlgh-1^−/−^* mice, the absence of the nasopharyngeal passage (NP), and the shortened snout and mandible (arrows). NC, nasal cavity; NP, nasopharyngeal passage; P, pharynx; T, tongue. Scale bar = 250 µm.

To understand the impact of the loss of *Dlgh-1* on mouse development, *Dlgh-1^+/+^*, *Dlgh-1^+/−^*, and *Dlgh-1^−/−^* mice were collected at embryonic stages E11.5, E14.5, E16.5, and E18.5. *Dlgh-1^−/−^* embryos were viable at all embryonic stages examined. However, at the gross level *Dlgh-1^−/−^* embryos exhibited an abnormal head shape that included a shortened snout (Figs. 2A, H–I). *Dlgh-1^−/−^* mice were smaller than their *Dlgh-1^+/+^* counterparts. This reduction in size was apparent overtly (Fig. 2A) and in histological section by E14.5 (Figs. 2B, C). At the gross anatomic level, *Dlgh-1^−/−^* mice also exhibited a secondary cleft-palate (Figs. 2D, E) that was consistent with the lack of a nasopharynx in these mutant animals observed in histological sections (Figs. 2F–I). All of these phenotypes of the *Dlgh-1^−/−^* mice were observed with 100% penetrance on both the FVB/NJ and C57BL/6J genetic backgrounds and were similar to those observed in the *Dlgh-1^gt/gt^* mice [34]. Additionally, the kidneys of the *Dlgh-1^−/−^* mice were smaller in size and exhibited a reduced tubule to mesenchyme ratio (data not shown), consistent with a defect in branching morphogenesis, which was reported for the previously described *Dlgh-1* mutant mice [35], [38]. Finally, defects in the ocular lens were also noted in the *Dlgh-1^−/−^* mice (data not shown) as reported previously for the *Dlgh-1^gt/gt^* mice [36] and mice in which *Dlgh-1* was specifically deleted in the lens [40]. Together, these data show that *Dlgh-1* is required for mouse survival, likely in part through its contribution to palate formation, and for multiple aspects of mouse development.

### Effect of Loss of *Dlgh-1* on Eyelid and Neural Tube Closure

**Figure 3:**
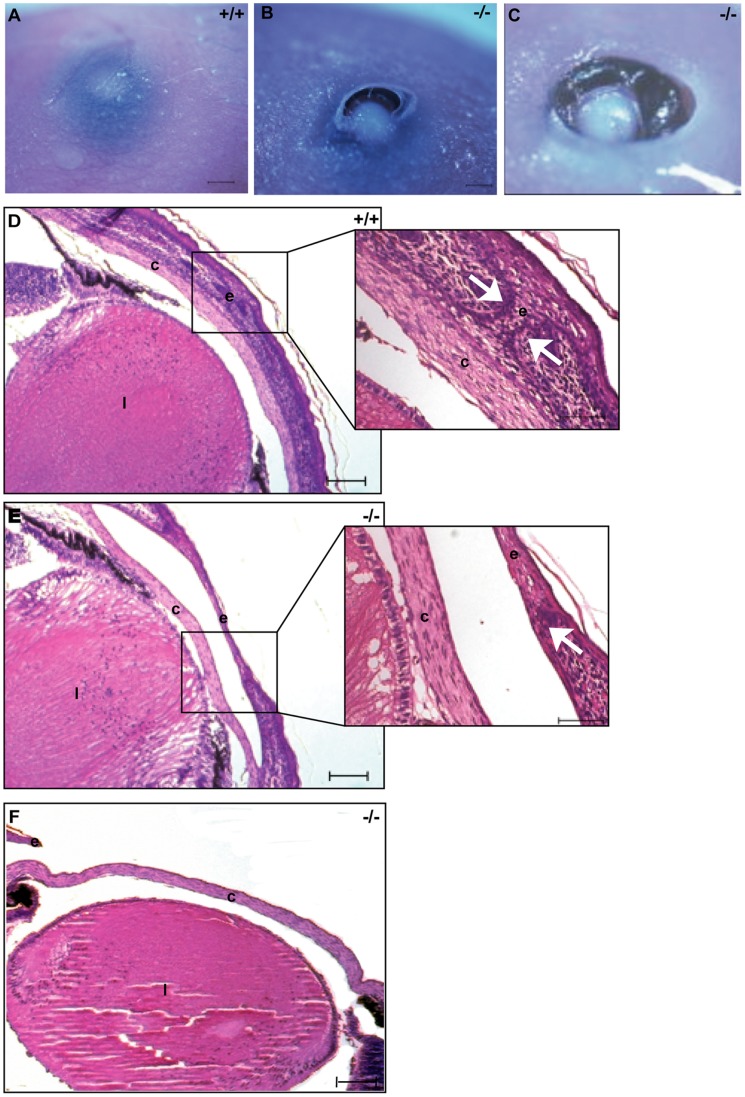
*Dlgh-1^−/−^* mice display defects in eyelid closure. E18.5 *Dlgh-^+/+−^* and *Dlgh-1^−/−^* mice on the C57BL/6 background were collected and analyzed for the presence of closed eyelids. *Dlgh-1^+/+^* mice (A) form closed eyelids while *Dlgh-1^−/−^* (B, C) littermates show defects in eyelid closure. (D–F) Hematoxylin and eosin staining of histological sections collected from E18.5 mice show completely closed eyelids in *Dlgh-1^+/+^* mice (D) whereas eyelids in *Dlgh-1^−/−^* mice were only partially closed (E) or completely open (F). Insets show a higher magnification of the eyelids. Arrows show the position of the tips of the dermis in *Dlgh-1^+/+^* (D) and *Dlgh-1^−/−^* (E) embryos, which failed to extend fully in the *Dlgh-1^−/−^* eye. Scale bar = 250 µm and 150 µm for insets. c, cornea; e, eyelids; l, lens.

**Figure 4:**
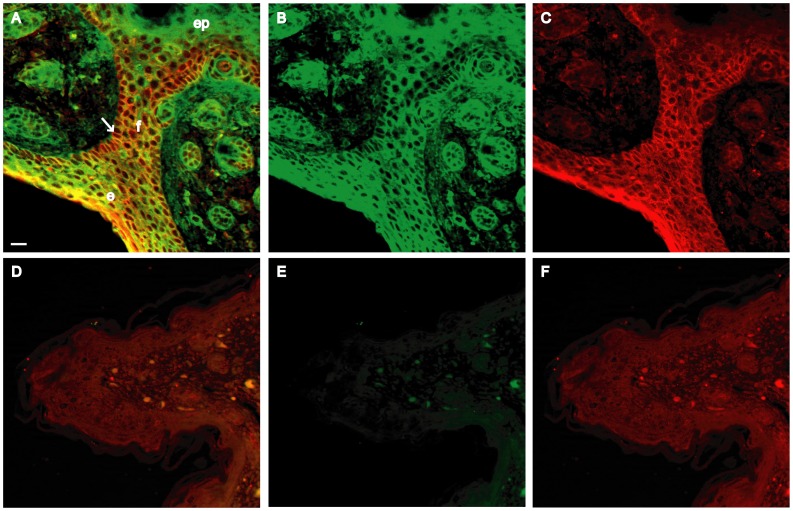
Expression of Dlgh-1 and Vangl2 proteins in the eyelid of *Dlgh-1^+/+^* and *Dlgh-1^−/−^* mice. Sections of sagittally oriented, paraffin embedded heads from newborn *Dlgh-1^+/+^* (A–C) and *Dlgh-1^−/−^* (D–F) mice on the C57BL/6J background were subjected to double immunofluorescence analysis with anti-Dlgh-1 (green) and anti-Vangl2 (red) antibodies. Shown are representative merged and unmerged images of the staining in the eyelid of *Dlgh-1^+/+^* (A–C) and *Dlgh-1^−/−^* (D–F) eyelids. Dlgh-1 (A, B) and Vangl2 (A, C) localized to the membranes of the epithelium (arrow in A) including the site of eyelid fusion, in *Dlgh-1^+/+^* mice. Staining for Dlgh-1 was absent in eyelid of the *Dlgh-1^−/−^* mice (E) and Vangl2 accumulated in the cytoplasm rather than in the membrane (D, F). e, epithelium; ep, epidermis; f, eyelid fusion. Scale bar = 50 µm.

The cleft palate and reduced size of the *Dlgh-1^−/−^* mice (Fig. 2) were observed on both the FVB/NJ or C57BL/6J backgrounds. However, some defects were noted exclusively on the C57BL/6J background. First, eyelid closure defects were apparent in 100% of C57BL/6J *Dlgh-1^−/−^* mice at E18.5 (Fig. 3). The severity of this phenotype varied. In some cases the epidermis had extended and fused; however, the dermis had not filled in (Fig. 3E). In other cases, the epidermis had failed to extend, leading to a complete failure of eyelid closure (Fig. 3F). To determine if the effect of Dlgh-1 deficiency on eyelid closure may be a direct effect, head sections from *Dlgh-1^+/+^* and *Dlgh-1^−/−^* mice were subjected to double immunofluorescence experiments using anti-Dlgh-1 and anti-Vangl2 antibodies (Fig. 4). Staining for Dlgh-1 was observed in the *Dlgh-1^+/+^* eyelid epithelium (Fig. 4A, B) but was absent in the *Dlgh-1^−/−^* eyelid (Fig. 4D, E), suggesting that the open eyelid defect may be a direct result of loss of Dlgh-1 function.

Interestingly, the failure of eyelid closure is also noted in the *circletail* (*Crc/Crc*) mouse, in which a defective Scrib protein lacking the C-terminal two PDZ domains is expressed [42], and in the *looptail* (*Vangl2^Lp/Lp^*) mouse, in which a defective Vangl2 protein carrying an amino acid substitution mutation (S464N) is expressed [43], [44]. The Vangl2 protein is normally expressed in the eyelid epithelium [43], [44], [45] and is primarily localized to the plasma membrane [45]. However, the mutant Vangl2 protein in *Vangl2^Lp/Lp^* mice fails to localize to the membrane; rather it is cytoplasmic [45]. Immunofluorescent staining of eyelids from *Dlgh-1^+/+^* mice showed that Vangl2 was tightly localized to the membranes in the eyelid epithelium (arrow, Fig. 4A, C) where there was overlap with Dlgh-1 staining. In contrast, Vangl2 was cytoplasmic in the eyelid of the *Dlgh-1^−/−^* mice (Fig. 4D, F). Thus, the correct subcellular localization of Vangl2 in the eyelid epithelium depends on *Dlgh-1*.

**Figure 5:**
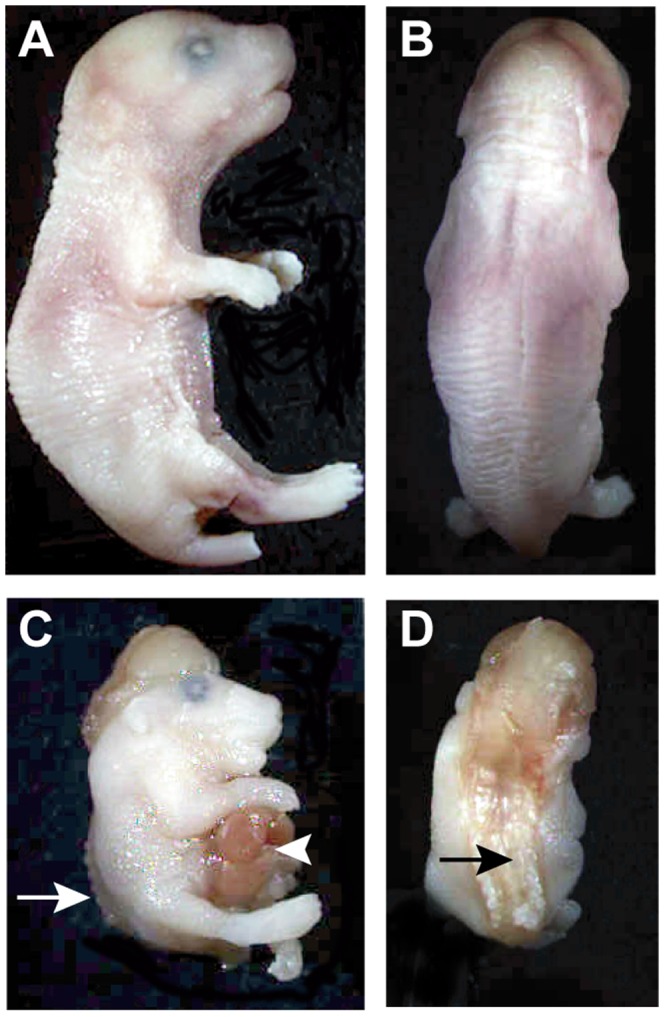
A fraction of *Dlgh-1^−/−^* mice display defects in neural tube closure. E16.5 *Dlgh-1^+/+^* and *Dlgh-1^−/−^* embryos on the C57BL/6J background were collected, examined for overt defects, and photographed under a dissecting microscope. Profile (A) and dorsal (B) views of an E16.5 *Dlgh-1* ^+/+^ embryo. Profile (C) and dorsal (D) views of an E16.5 *Dlgh-1^−/−^* embryo. In addition to craniorachischisis (C, D arrows), the *Dlgh-1^−/−^* embryo exhibits gastroschisis (C, arrowhead) and a rightward kink to the body axis (D).

*Vangl2^Lp/Lp^* and *Crc/Crc* mice also exhibit craniorachischisis [42], [44], [46], a severe neural tube closure defect in which the neural tube is open from the midbrain-hindbrain boundary to the tail. This same neural tube defect was observed in C57BL/6J *Dlgh-1* ^−/−^ mice at both E16.5 (Figs. 5C, D) and E18.5 (not shown). Unlike the open eyelid phenotype, however, craniorachischisis occurred with low penetrance; only 6.7% (2 out of 30) of the C57BL/6J *Dlgh-1* null mice displayed this defect. *Dlgh-1^−/−^* mice that exhibited craniorachischisis also exhibited gastroschisis, an externalization of the gut (Fig. 5A, C). Closure of the eyelid and neural tube involves a process of polarized cell movement known as convergent extension [3], [47]. Thus, the defects in eyelid and neural tube closure exhibited in the *Dlgh-1* ^−/−^ mice suggest a new role for *Dlgh-1* in the mouse in the regulation of convergent extension.

### Effect of the Loss of *Dlgh-1* on Cochlear Hair Cell Stereociliary Bundle Orientation

Recent studies in vertebrates have shown that convergent extension is regulated, at least in part, by a group of proteins that are involved in planar cell polarity (PCP) in *Drosophila* [3]. PCP refers to the polarization of cells within the plane of a cell sheet. In *Drosophila*, PCP regulates wing hair, body bristle, and eye ommitidial cluster orientation. In mammals, the uniform orientation of stereociliary bundles of the hair cells within the cochlea represents a distinctive example of PCP. In *Vangl2^Lp/Lp^* mice, the stereociliary bundles in the inner (IHC) and outer (OHC) hair cell layers are misoriented [4]. Similarly, in *Crc/Crc* mice the stereociliay hair bundles in the second and third row of OHCs are misoriented [4], Together, these data identify a role for *Vangl2* and *Scrib* in the regulation of the PCP pathway in vertebrates.

**Figure 6:**
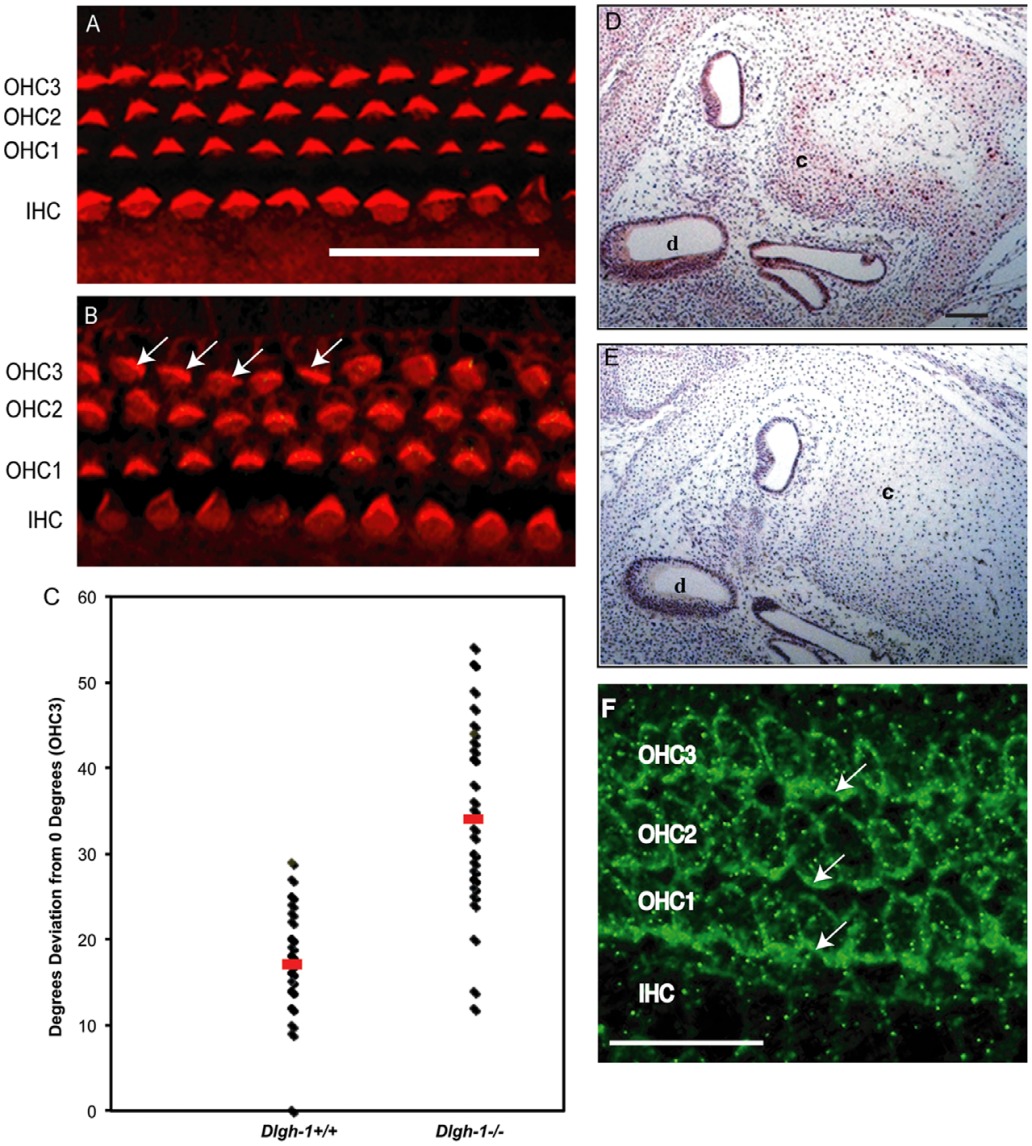
*Dlgh-1^−/−^* mice display defects in outer cell hair polarity. (A, B) Representative images of the OHC and IHC layers in the cochlea of *Dlgh-1^+/+^* and *Dlgh-1^−/−^* mice. The organ of Corti was isolated from the cochleae of E18.5 *Dlgh-1^+/+^* (A) and *Dlgh-1* ^−/−^ (B) embryos, stained with phalloidin to visualize the actin bundles of the inner and outer hair cells, and viewed by confocal microscopy. Arrows indicate cells with misoriented stereociliary bundles in OHC3 row of the *Dlgh-1^−/−^* mice. (C) Measurement of misorientation of sterociliary bundles in OHC3 layer of *Dgh-1^+/+^* and *Dlgh-1^−/−^* mice. The angle of sterociliary bundles in OHC3 of *Dlgh-1^+/+^* and *Dlgh-1^−/−^* mice was measured in reference to a line parallel to the neural-abneural axis and perpendicular to the plane of the pillar cells. Plotted is the deviation in degrees from the neural-abneural axis for each OHC3 bundle measured in (4 cochleae from 3 mice of each genotype, 36 cells for *Dlgh-1^+/+^* and 33 cells for *Dlgh-1^−/−^* mice). No deviation from the neural-abneural axis was assigned a value of 0°. The average angle deviation from 0° for OHC3 bundles in *Dlgh-1^−/−^* cochlea (33.7°+/−1.869, red bar) was significantly higher (p = 1.5×10^−9^) than that of the *Dlgh-1^+/+^* (17.3°+/−0.869, red bar). (D, E) Immunohistochemical detection of Dlgh-1 protein in the cochlea of E15.5 mouse embryos. Transverse sections of paraffin embedded heads from control *Dlgh-1^+/+^* mice were subjected to immunohistochemistry using (D) an anti-Dlgh-1 specific antibody and alkaline phosphatase detection or (E) no primary antibody. The pink color in the epithelium of the cochlear duct and cartilage in (D) is indicative of the presence of Dlgh-1 protein in these structures. (F) Immunofluorescent detection of Dlgh-1 protein in the cochlear hair cells of P2 mice control. Fixed cochlear explants from *Dlgh-1^+/+^* mice were subjected to immunofluorescence using an anti-Dlgh-1 (green) antibody. Arrows show concentration of Dlgh-1 on the basal-lateral membranes of the OHC cells. IHC, inner hair cells, OHC1, 2, 3, outer hair cell rows 1, 2, and 3. c, cartilage in cochlea, d, cochlear duct. Scale bar = 50 µm.

Because *dlg* and *scrib* are known to interact in *Drosophila* [2] and because the open eyelid, neural tube, and gut phenotypes of the *Dlgh-1^−/−^* embryos (Fig. 4) resemble that of *Crc/Crc* mutant mice [42], we asked if stereociliary bundle orientation was affected in the *Dlgh-1* null mice. Cochlear sensory epithelia (organs of Corti) from E18.5 *Dlgh-1^+/+^* and *Dlgh-1^−/−^* embryos were stained with phalloidin to label hair cell sterocilia, a structure rich in filamentous actin, and the specimens analyzed by confocal microscopy. Normally, the stereociliary bundles are arranged uniformly around the primary cilia and oriented toward the outer edge of the organ. The IHC bundles are arranged in a curved shape while the OHC bundles are arranged in a v-shape [48]. The stereociliary bundles in the IHC row of the *Dlgh-1^−/−^* mice were uniformly polarized, as in the *Dlgh-1^+/+^* mice (Fig. 6A, B). The stereociliary bundles in all three rows of OHCs of the *Dlgh-1^+/+^* mice were uniformly oriented (Fig. 6A). However, many stereociliary bundles in the third row of OHCs in the *Dlgh-1^−/−^* cochleae were misoriented (Fig. 6B, arrows). Occasionally, stereociliary bundles in the second OHC layer were also misoriented (Fig. 6B). The angle of orientation of individual stereociliary bundles in the OHC3 layer relative to the neural-abneural axis in *Dlgh-1^+/+^* and *Dlgh-1^−/−^* cochlea were measured and the degrees of deviation from this axis calculated (Fig. 6C). The average degrees of deviation for bundles in the *Dlgh-1^−/−^* mice (33.7+/−1.87 degrees) was significantly higher (p = 1.5×10^−9^) than in the *Dlgh-1^+/+^* mice (17.3+/−0.87 degrees). To determine if the effect of *Dlgh-1* deficiency on the cochlea might be a direct effect, head sections from day E15.5 *Dlgh-1^+/+^* embryos were immunostained with an anti-Dlg-1 antibody (Fig. 6D). Immunoreactivity was observed in the epithelial cells of the duct, which give rise to the sensory cells, and cartilage within the cochlea, indicating the presence of Dlgh-1 protein in these structures. To further determine if the effect of Dlgh-1 deficiency on hair cell orientation might be a direct effect, cochlear explants from P2 control mice were subjected to immunofluorescence for Dlgh-1. Immunoreactivity was observed in the basal-lateral membrane domain of the outer hair cells (Fig. 6F, arrows). The expression of Dlgh-1 in tissues such as the eyelid and hair cells, which show defects in the *Dlgh-1^−/−^* mouse, together with the open eyelid, mislocalization of PCP protein Vangl2 in the open eyelid, craniorachischisis, and misorientation of cochlear stereociliary in the *Dlgh-1^−/−^* mice (Figs. 3, 4, 5, 6) suggest a role for *Dlgh-1* in the regulation of PCP in the mouse.

### Effect of the Loss of *Dlgh-1* in Skeletogenesis

**Figure 7:**
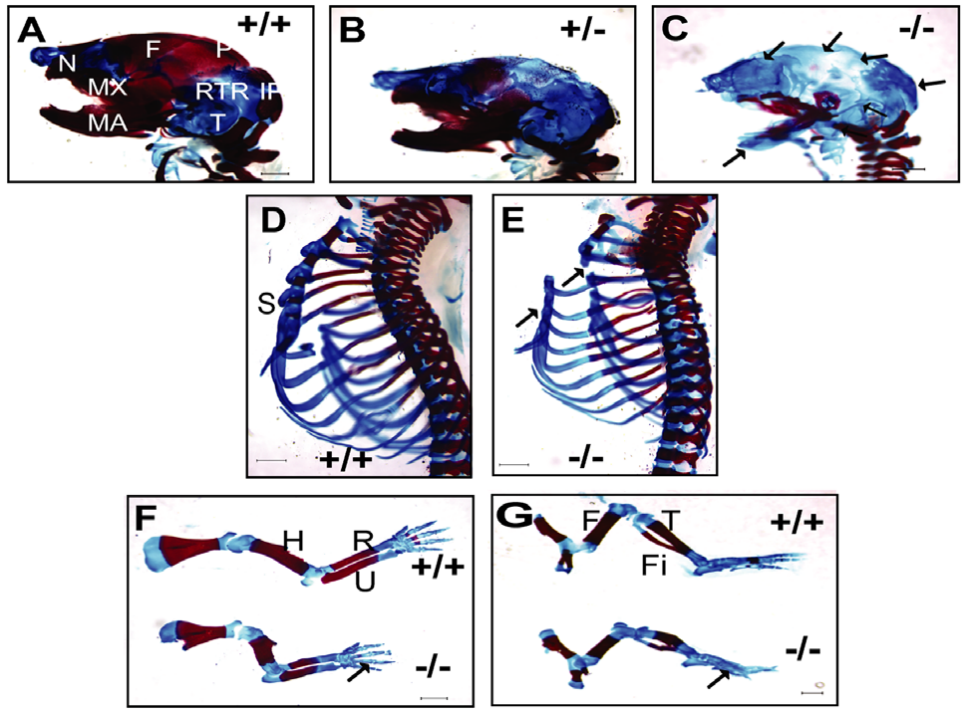
*Dlgh-1^−/−^* mice display defects in skeletogenesis. Eviscerated carcasses of E18.5 *Dlgh-1^+/+^*, *Dlgh-1^+/−^*, and *Dlgh-1^−/−^* mice on the FVB/NJ background were prepared and stained for cartilage and bone with alcian blue and alizarin red, respectively. Craniofacial skeleton in (A) *Dlgh-1^+/+^,* (B) *Dlgh-1^+/−^*, and (C) *Dlgh-1^−/−^* mice. Arrows show loss of bone formation in *Dlgh-1^+/−^* and *Dlgh-1^−/−^* mice. F, frontal bone; IP, intraparietal bone; P, parietal bone; N, nasal; Ro, rostral bone; RTP, retrotemporal process; T, temporal bone. (D–G) Axial structures of *Dlgh-1^+/+^* (D) and *Dlgh-1^−/−^* mice (E). Forelimb (F) and hindlimb (G) structures. Arrows show the absence of bone formation in *Dlgh-1^−/−^* mice. S, sternebrae; H, humerus; R, radius; U, ulna; F, femur; Fi, fibula; T, tibia. Scale bar in A-E = 1000 µm, in F = 650 µm and in G = 500 µm.

The *Dlgh-1* ^gt/gt^ mice exhibited a cleft palate and a hypotrophic mandible [34], suggesting that *Dlgh-1* may be required for the formation of at least certain craniofacial bones. To test this hypothesis, the skeletal frames of E18.5 *Dlgh-1^+/+^, Dlgh-1^+/−^,* and *Dlgh-1^−/−^* mice on both the FVB/NJ and C57BL/6J background were stained with alizarin red and alcian blue to visualize bone and cartilage, respectively. *Dlgh-1^−/−^* mice on both the FVB/NJ (Fig. 7) and C57BL/6J (not shown) genetic backgrounds had shorter mandibles (MA) and maxillas (MX), which was consistent with the short snout phenotype. However, uniquely on the FVB/NJ background, *Dlgh-1^−/−^* mice also showed additional skeletal defects. *Dlgh-1^−/−^* embryos exhibited reduced ossification of multiple craniofacial structures including the nasal bone (N), the retrotympanic process (RTP), and the tympanic bone (T) (Figs. 7A, C). Interestingly, the developing calvarium was also affected as the ossification of the frontal bone (F) and the parietal bone (P) were reduced or absent in the *Dlgh-1^−/−^* mice (Fig. 7C). Furthermore, the parietal bone was absent in one *Dlgh-1^+/−^* specimen on the FVB/NJ background, suggesting a dosage-dependent effect of Dlgh-1 on calvarium development (Fig. 7B).

The skeletal defects in the *Dlgh-1^−/−^* mice on the FVB/NJ background extended to a reduction in the ossification of the ribs, as well as the second, third, and fourth sternebrae (S) (Fig. 7E). The long bones of the fore- and hindlimbs were also shortened by approximately 20% and 18%, respectively, in the *Dlgh-1^−/−^* mice when compared to their *Dlgh-1^+/+^* counterparts (Figs. 7F, G). Calcification in the phalanges, while apparent in the limbs of the *Dlgh-1^+/+^* mice, was absent in the limbs of the *Dlgh-1^−/−^* mice (Figs. 6F, G, arrow). Taken together, these results suggest that *Dlgh-1* is required for proper skeletogenesis of craniofacial, axial and long bones. Furthermore, as the calvarium forms through intramembranous bone formation whereas the other craniofacial bones and the axial bones form through endochondral ossification [49], these data show that both forms of bone development are affected when *Dlgh-1* is ablated in the mouse.

## Discussion

The MAGUK protein Dlgh-1, which is highly conserved cross-species, has emerged as an important factor in the regulation of cell-cell adhesion, apical-basal polarity, and cell proliferation in *Drosophila* [1], [2]. The regulation of these cellular aspects is critical for the establishment of specialized organs during embryogenesis. In the current study, we assess the role of *Dlgh-1* in mouse embryogenesis (see Table 1 for a summary of phenotypes). We provide evidence that loss of *Dlgh-1* affects PCP during mouse embryogenesis. We further provide evidence that loss of *Dlgh-1* causes defects in skeletal structures arising through both endochondral and intramembranous ossification. These findings demonstrate newly discovered roles for *Dlgh-1* in vertebrate development and suggest that the *Dlgh-1^−/−^* mouse may serve as an animal model to study major congenital birth defects in humans such as defects in neural tube closure and skeletal malformations.

**Table 1:** Summary of Phenotypes in *Dlgh-1* Null Mice.

Defect	Mouse Strain	Penetrance	Reported in *Dlg^gt^* mice
Perinatal lethality	Both	100%	Yes
Cleft palate	Both	100%	Yes
Shortened snout	Both	100%	Yes
Reduced size	Both	100%	Yes
Eyelid closure	C57BL/6J	100%^a^	No
Neural tube closure	C57BL/6J	6.7%	No
Cochlear hair cells	C57BL/6J	100%	No
Skeletal^b^	FVB/NJ	100%	No

a Eyelid closure defects were observed in 100% of *Dlgh-1^−/−^* mice on the C57BL/6J background. However, the severity of the phenotype varied from animal to animal.

b Defects in bone formation were observed throughout the head, trunk and limbs of the *Dlgh-1^−/−^* embryos on the FVB/NJ background.

### Role of *Dlgh-1* in Mouse Organogenesis

The results presented in this study suggest that *Dlgh-1* plays a vital role during mouse organogenesis. Defects in the mandible, the kidneys, and the lens have been previously described in the *Dlgh-1^gt/gt^* mice [34], [35], [36]. The formation of these structures and organs was likewise affected in our *Dlgh-1* null mice (Fig. 2 and data not shown). More recently, novel defects in the ureteric smooth muscle and the urogenital tract have been observed in *Dlgh-1^−/−^* mice [38], [39]. In the present study, we provide evidence for *Dlgh-1* playing a role in the skeletogenesis of trunk and limb structures, in neural tube and eyelid closure, and in the organization of the stereociliary bundles in the cochlea. These latter phenotypes were not reported previously in mice carrying the gene trap insertion in *Dlgh-1* [34] and we have not observed open eyelids or open neural tubes in C57BL/6 *Dlgh-1^gt/gt^* mice in our laboratory (M.M. Nguyen, C. Rivera, and A.E. Griep, unpublished observations). As the N-terminal portion of Dlgh-1 including the L27 and three PDZ domains are intact in the Dlgh-1^gt^ fusion protein [34], [37], it is likely that the function of one or more of these protein-protein interaction domains is required for Dlgh-1′s role in the novel phenotypes we have observed.

### Role of *Dlgh-1* in Planar Cell Polarity

Planar cell polarity is the mechanism through which the orientation of cells is coordinated within the plane of a cell sheet. In vertebrates, PCP has been suggested to be required for several developmental processes that involve convergent extension, such as lengthening of the body axis, eyelid closure, neural tube closure, branching morphogenesis in the kidney and lung, and the sterociliary cell hair bundle orientation in cochlea [3]. *Dlgh-1^−/−^* mice, with variable penetrance, exhibited a reduced body length to width ratio, open eyelids (Fig. 3), craniorachischisis (Fig. 5), and misorientation of stereocilia in the third row of cochlear OHCs (Fig. 6). Moreover, Dlgh-1 is expressed in the eyelid epithelium (Fig. 4A, B) and cochlear hair cells (Fig. 6F) and *Dlgh-1* deficiency led to mislocalization of the core PCP protein, Vangl2, in the mutant eyelid (Fig. 4D, F). Thus, this study provides the first evidence in support of a role for *Dlgh-1* in the regulation of PCP in the mouse *in vivo.*

Although much remains to be learned about the molecular and genetic mechanisms regulating PCP, from studies in the *Drosophila* eye and wing, factors including Frizzled (Fz), Dishevelled (Dsh), Strabismus/Van Gogh (stbm/Vang), and Starry night/Flamingo (Stan/Fmi) are thought to mediate PCP through non-canonical Wnt signaling [3]. This pathway appears to be highly conserved in vertebrates. Wnt signaling has been shown to be required for mediating the organization of cochlear OHCs into their distinctive unidirectional orientation during development [48], [50]. *Fz3* ^−/−^;*Fz6* ^−/−^, *Dvl1^−/−^;Dvl2^−/−^*, and *Lp/Lp* mice show reduced body length to width ratio, craniorachischisis, failure of eyelid closure, and misorientation of stereociliary bundles at the apices of sensory hair cells [4], [42], [51], [52], [53] Additionally, this small group of factors in vertebrates includes Celsr1 [54], a protocadherin and homolog of *Stan/Fmi*, and PTK 7, a protein tyrosine kinase [55].

Interestingly, Fz3, Fz6, and Vangl2 contain C-terminal PDZ binding motifs whereas Dvl1 and Dvl2 are PDZ domain proteins, suggesting that PDZ domain proteins and their ligands interact to play a major role in PCP. *Dvl2* genetically interacts with *Vangl2* and Vangl2 is required for the proper membrane localization of Dvl2 in the cochlear hair cells [52]. Vangl2 interacts with Fz3 and is required for targeting of Fz3 to the membrane of the cochlear hair cells [56]. It has recently been shown that these core PCP proteins also interact with other PDZ proteins in vertebrate systems. Fz4 and Fz7 have been shown to interact with the MAGUK protein, MAGI-3, through the PDZ binding motif of Fz and the first PDZ domain of MAGI-3 *in vitro* and in epithelial cells [57] and Dlgh-1 has been shown to interact through its PDZ domain with the C-terminal PDZ binding motif in Fz1, Fz4, and Fz7 at least in yeast two hybrid experiments [58], [59]. Mice defective for the PDZ protein Scrib (*Crc/crc* mice) exhibit the same phenotypes as mouse *Fz*, *Dvl*, and *Vangl2* mutants. *Scrib* been shown to genetically interact with *Vangl2* to modulate PCP in the cochlea [4] and Scrib binds to Vangl2 [56]. In this study, we have shown that the phenotype of *Dlgh-1* null mice bears many similarities to the phenotypes of PCP mutant mice. Furthermore, we have shown that *Dlgh-1* is expressed in the eyelid epithelium (Fig. 4A, B) and the outer hair cells of the cochlea (Fig. 6F) and is required for correct subcellular localization of Vangl2 in the eyelid (Fig. 4D, F). Therefore, we suggest that Dlgh-1 also is a member of this group of factors involved in the regulation of PCP in the mouse, which is has very recently been shown to include another mouse Dlg, Dlg-3 [60]. The role for Dlgh-1 in PCP in properly localizing Vangl2 to the membrane may be a critical function for PCP as Vangl2 has been shown to be required for proper localization of Fz3 [56] and Dvl2 [61], [62].

We observed differences in the phenotypes of *Dlgh-1^−/−^* embryos depending on the genetic background. On the C57BL/6J background, eyelid closure defects were present in 100% and craniorachischisis was present in 6.7% of *Dlgh-1^−/−^* embryos (Figs. 3, 5). Neither of these phenotypes was observed in *Dlgh-1^−/−^* embryos on the FVB/NJ background. The low penetrance of craniorachischisis in *Dlgh-1^−/−^* embryos on the C57BL/6J background is similar to observations of low penetrance of craniorachischisis in *Dvl1^−/−^;Dvl2^−/−^* mice on the C57BL/6J background. By contrast the penetrance of craniorachischisis is high on the 129 background [52]. The observation that the penetrance of some phenotypes associated with PCP is dependent on mouse genetic background suggests either that there are additional genetic modulators of PCP that have yet to be identified or that polymorphisms in one or more of the already identified genes on these different genetic backgrounds influences PCP.

### Role of *Dlgh-1* in Skeletogenesis

The cleft palate and hypotrophic mandible in the *Dlgh-1^gt/gt^* mice indicated that this PDZ domain protein might be important in the development of craniofacial skeletal structures [34]. The *Dlgh-1^−/−^* mice not only recapitulated the craniofacial defects observed in the *Dlgh-1^gt/gt^* mice, but also displayed reduced ossification of the maxilla, middle ear, frontal, and parietal bones and overall reduced size of the skeleton (Fig. 7).

One mechanism through which loss of *Dlgh-1* may affect craniofacial development is through disruption of WNT/PCP signaling [63]. Recently, it has been shown that certain core Wnt/PCP proteins are expressed in cranial neural crest (CNC) cells [64], [65], which give rise to most of the skeletal structures of the head and neck, and these core PCP proteins are required for CNC migration [66]. As noted above, Dlgh-1 has been shown to bind to several Fz receptors, including those commonly associated with Wnt/PCP signaling [58], [59]. Likewise, Wnt5a, which has been suggested to be a regulator of Wnt/PCP signaling in the mouse [50], is expressed in outgrowing regions of the facial primordia and *Wnt5a* mutant mice display abnormally shaped head and shortened snout and mandible [67]. Finally, *Wnt5a* deficiency in mice results in cleft palate formation due to inhibition of the directional migration of cells that is required for palate formation [68]. Thus, the similarity of the craniofacial defects in *Wnt5a^−/−^*, *Vangl2^Lp/Lp^*, and *Dgh-1^−/−^* mice suggests that *Dlgh-1* may be a regulator of craniofacial skeletogenesis through a mechanism that involves modulating Wnt/PCP.

The *Dlgh-1^−/−^* mice exhibited defects not only in skeletal structures of the head, but also of the trunk and limbs and the skeletons were reduced in size overall. In particular, the forelimbs and hindlimbs of *Dlgh-1^−/−^* mice were approximately 20% shorter than those of *Dlgh-1^+/+^* mice and the humerus was clearly wider in the mutants as compared to controls (Fig. 6). One possibility is that the reduced size is due to an inhibition of proliferation, as Dlgh-1 has been associated with regulating proliferation [22]. However, Wnt/PCP also has been shown to play a role in limb development [69], [70]. *Wnt5a* mutant mice exhibit truncated limbs [67] and the directional outgrowth of the limb has been shown to require Wnt5a/JNK signaling in the distal mesenchyme [71], [72]. Similarly, *Vangl2^Lp/Lp^* mice display shorter and wider limbs [73]. Recent studies have demonstrated a genetic and mechanistic link between Wnt5a and Vangl2 through the frizzled-like receptor, Ror2 [73], which binds Wnt5a [74], clearly establishing a connection between Wnt5a and a core PCP protein. Thus, the similarities in the limb phenotypes of these mouse mutants and the *Dlgh-1^−/−^* mice combined with the known mechanistic link between Dlgh-1 and Vangl2 suggest that the mechanism through which *Dlgh-1* exerts its effects may be through modulating Wnt/PCP.

Although, the most likely explanation for the defects in skeletogenesis in the *Dlgh-1* mutant mice is that *Dlgh-1* is required the Wnt/PCP-dependent development of these skeletal structures, analysis of mouse mutants has demonstrated that numerous other signaling pathways play a role in skeletogenesis. These include FGFR [75], [76], TGFβII [75], [77], BMP [78], and Wnt/β-catenin [79], [80], [81], [82]. Further studies will be required to determine if the mechanism through which *Dlgh-1* affects bone formation is through an effect on Wnt/PCP and how Wnt/PCP signaling coordinates with these other signaling pathways to direct the proper formation of the skeleton.

In summary, we provide evidence of new roles for *Dlgh-1* in the regulation of PCP and both endochondral and intramembranous bone formation in the mouse.

## Materials and Methods

### Ethics Statement

All procedures using mice conformed to the Guide for the Care and Use of Laboratory Animals of the National Institutes of Health and the ARVO Statement for the Use of Animals in Ophthalmic and Vision Research. The protocol covering these studies (protocol #M00712) was approved by the Institutional Animal Care and Use Committee of the University of Wisconsin School of Medicine and Public Health (Animal Welfare Assurance #A3368-01).

### Generation of the *Dlgh-1* Null Mouse

The generation of the mice carrying the gene targeted *Dlgh-1* conditional allele (Fig. 1A) has been described in detail previously (Rivera et al., 2009). To generate mice carrying a germline null mutation in *Dlgh-1*, female *Dlgh-1^fn/+^* mice were mated to male *EIIA-Cre* transgenic mice [41] (Fig. 1). Removal of exon 8, which encodes a portion of the first PDZ domain of the Dlgh-1 protein, results in a frameshift mutation that generates a termination codon 3 codons downstream of the deletion. Male F1 progeny were mated to stock C57BL/6J or FVB/NJ females. Tail DNAs from the F2 progeny were screened by PCR for both the WT allele and the null allele using the Dlgh-1 5′ primer CATCATGGTTGAAGTGCTCTGGGC paired to the Dlgh-1 3′ primer GGAAGGAAACTCACGGATGGTCC (Fig. 1).

### Gross and Microscopic Analyses

For examination of morphological defects at the gross level**,** newborn mice were examined for overt defects and photographed under a dissecting microscope. To examine palate formation, E18.5 mice were collected from pregnant dams and an incision made through the temporal-mandibular joint to expose the palate surface. Embryos were staged by designating the morning of the vaginal plug as day E0.5.

For histological analysis, E14.5, E16.5, and E18.5 embryos and neonates from *Dlgh-1^+/+^, Dlgh-1* ^+/−^, and *Dlgh-1^−/−^* animals were fixed in 4% paraformaldehyde at 4°C for 2–3 days, dehydrated through graded alcohols and xylenes, and embedded in paraffin for transverse, coronal, and sagittal sectioning. E18.5 day embryos were decalcified prior to dehydration. Serial 5 µm paraffin sections were stained with hematoxylin and eosin and viewed by light microscopy. For detection of Dlgh-1 protein by immunohistochemistry, transverse oriented sections from E15.5 embryos were rehydrated and stained with an anti-SAP97 antibody (1∶500 dilution, obtained from J. Hell, University of Iowa), followed by Vectastain ABC-AP and Vector Red (Vector Labs) according to manufacturer’s instructions. For detection of Dlgh-1 and Vangl2 proteins by immunofluorescence, sagittal sections from paraffin embedded newborn *Dlgh-1^+/+^* and *Dlgh-1^−/−^* mice were rehydrated, boiled in a rice cooker for 30 minutes in a solution of 10 mM Tris/1 mM EDTA/0.05% Tween-20 pH 9.0, blocked in 5% donkey serum diluted in 1X PBS, incubated with an anti-Dlgh-1 antibody (1∶1000 dilution, catalog number NBP1-48054, Novus Biologicals) and an anti-Vangl2 antibody (1∶100 dilution, catalog number sc-46560, Santa Cruz Biotechnology) overnight at 4°C. The next day, sections were washed and incubated with fluorescein conjugated horse anti-mouse and Alexa Fluor 568 conjugated goat anti-rabbit secondary antibodies for 1 hour at room temperature. Stained sections were viewed on a Zeiss Axioimager M2 microscope and images captured using Axiovision 4.8.2 software.

### Cochlear Hair Cell Stereociliary Bundle Staining

The intact organ of Corti from E18.5 *Dlgh-1^+/+^, Dlgh-1* ^+/−^, and *Dlgh-1^−/−^* embryos was isolated in Hank’s Balanced Salt Solution (HBSS, Gibco, Invitrogen) and fixed in 4% paraformaldehyde for 3 hours at 4°C. After initial fixation, the stria vascularis and tectorial membrane were removed and then the explants were fixed in 4% paraformaldehyde for overnight at 4°C. The fixed explants were permeabilized with 0.5% triton-X100 in HBSS for 30 minutes at room temperature, then stained with Alexa Fluor 568-phalloidin (1 µg/ml, Molecular Probes) for 1 hour, and finally mounted as a surface preparation on a glass slide. Stained sections were viewed on a Nikon Diaphot 200 confocal microscope and images were captured using BioRad 1024 software. The orientation of individual stereociliary bundles was determined relative to a line parallel to the neural-abneural axis and perpendicular to the row of pillar cells, as described by Montcouquiol et al. [4]. The deviation from the neural-abneural axis was measured in degrees with a protractor. A cell with the vertex of the sterociliary bundle parallel to the neural-abneural axis was assigned 0^0^ deviation. A total of 4 cochleae from 3 different mice of each genotype were analyzed. The data were subjected to statistical analysis using the two-sided Wilcoxon Rank Sums test and p<.05 was considered statistically significant. For detection of Dlgh-1 in the hair cells by immunofluorescence, fixed explants were immersed in 150 mM Tris-HCl, pH 9.0 at 65°C for 15 minutes followed by acetone treatment at −20°C for 20 minutes. After washing, explants were blocked in 10% serum diluted in 0.5% Triton X-100 in PBS for 2 hours at room temperature. Explants then were incubated with anti-Dlgh-1 antibody (1∶500 dilution, Novus Biologicals) overnight at 4°C followed by incubation with fluorescein conjugated horse anti-mouse secondary antibody for 2 hours at room temperature. Immunostained explants were viewed and photographed as described above.

### Immunoblot Analysis

Whole tissues and MEFs were lysed in RIPA Buffer [1% NP-40, 0.1% sodium dodecyl sulfate (SDS), 0.5% deoxycholatate sodium (DOC), 150 mM NaCl, 50 mM Tris, pH 8.0] and centrifuged at 13,200 rpm for 15 minutes at 4°C. Protein concentrations in cell extracts were determined by the bicinchoninic acid (BCA) assay. Fifty µg of RIPA-soluble lysate from E18.5 *Dlgh-1^+/+^, Dlgh-1* ^+/−^, and *Dlgh-1^−/−^* animals were run on a 7.5% acrylamide gel and transferred to a PVDF membrane. The blots were blocked in 5% nonfat dry milk 1X PBS containing 0.1% Tween-20 and then incubated with mouse anti-Dlgh-1 (catalog number 610874, BD Transduction Laboratories), which was raised against amino acids 5–213 of human Dlgh-1 (amino acids upstream of the first PDZ domain), for 3 hours at room temperature in block solution. After washing and incubation with HRP-coupled goat-mouse (Pierce), detection was performed with ECL plus (Amersham). Blots were reprobed with mouse anti-β-actin (Sigma) as loading control.

### Skeletal Staining

Eviscerated carcasses from E18.5 *Dlgh-1^+/+^, Dlgh-1* ^+/−^, and *Dlgh-1^−/−^* animals were fixed in 95% ethanol for 5 days at room temperature. After fixation, samples were cleared in 1% KOH for 2 days at RT. Samples were stained in 0.5% alcian blue 8GS (in 70% ethanol, 5% glacial acetic acid) and 0.2% alizarin red S solution (95% ethanol, 5% glacial acetic acid) for 5 days at room temperature. Samples were destained in 1% KOH for 2 days at room temperature and in graded glycerol/1% KOH at room temperature.

## References

[1] Woods DF, Hough C, Peel D, Callaini G, Bryant PJ (1996) Dlg protein is required for junction structure, cell polarity, and proliferation control in Drosophila epithelia. J Cell Biol 134: 1469–1482. 8830775 10.1083/jcb.134.6.1469 PMC2120992

[2] Bilder D, Li M, Perrimon N (2000) Cooperative regulation of cell polarity and growth by Drosophila tumor suppressors. Science 289: 113–116. 10884224 10.1126/science.289.5476.113

[3] Wang Y, Nathans J (2007) Tissue/planar cell polarity in vertebrates: new insights and new questions. Development 134: 647–658. 17259302 10.1242/dev.02772

[4] Montcouquiol M, Rachel RA, Lanford PJ, Copeland NG, Jenkins NA, et al (2003) Identification of Vangl2 and Scrb1 as planar polarity genes in mammals. Nature 423: 173–177. 12724779 10.1038/nature01618

[5] Bossinger O, Klebes A, Segbert C, Theres C, Knust E (2001) Zonula adherens formation in Caenorhabditis elegans requires dlg-1, the homologue of the Drosophila gene discs large. Dev Biol 230: 29–42. 11161560 10.1006/dbio.2000.0113

[6] Firestein BL, Rongo C (2001) DLG-1 is a MAGUK similar to SAP97 and is required for adherens junction formation. Mol Biol Cell 12: 3465–3475. 11694581 10.1091/mbc.12.11.3465 PMC60268

[7] Koppen M, Simske JS, Sims PA, Firestein BL, Hall DH, et al (2001) Cooperative regulation of AJM-1 controls junctional integrity in Caenorhabditis elegans epithelia. Nat Cell Biol 3: 983–991. 11715019 10.1038/ncb1101-983

[8] McMahon L, Legouis R, Vonesch JL, Labouesse M (2001) Assembly of C. elegans apical junctions involves positioning and compaction by LET-413 and protein aggregation by the MAGUK protein DLG-1. J Cell Sci 114: 2265–2277. 11493666 10.1242/jcs.114.12.2265

[9] Segbert C, Johnson K, Theres C, van Furden D, Bossinger O (2004) Molecular and functional analysis of apical junction formation in the gut epithelium of Caenorhabditis elegans. Dev Biol 266: 17–26. 14729475 10.1016/j.ydbio.2003.10.019

[10] Bilder D (2001) PDZ proteins and polarity: functions from the fly. Trends Genet 17: 511–519. 11525834 10.1016/s0168-9525(01)02407-6

[11] Bellaiche Y, Radovic A, Woods DF, Hough CD, Parmentier M-L, et al (2001) The Partner of Inscuteable/Discs-large complex is required to establish planar polarity during asymmetric cell division in Drosophila. Cell 106: 355–366. 11509184 10.1016/s0092-8674(01)00444-5

[12] Caruana G (2002) Genetic studies define MAGUK proteins as regulators of epithelial cell polarity. Int J Dev Biol 46: 511–518. 12141438

[13] Lin L, Sahr KE, Chishti AH (1997) Identification of the mouse homologue of human discs large and rat SAP97 genes. Biochim Biophys Acta 1362: 1–5. 9434093 10.1016/s0925-4439(97)00059-8

[14] Lue RA, Marfatia SM, Branton D, Chishti AH (1994) Cloning and characterization of hdlg: the human homologue of the Drosophila discs large tumor suppressor binds to protein 4.1. Proc Natl Acad Sci U S A 91: 9818–9822. 7937897 10.1073/pnas.91.21.9818 PMC44908

[15] Muller BM, Kistner U, Veh RW, Cases-Langhoff C, Becker B, et al (1995) Molecular characterization and spatial distribution of SAP97, a novel presynaptic protein homologous to SAP90 and the Drosophila discs-large tumor suppressor protein. J Neurosci 15: 2354–2366. 7891172 10.1523/JNEUROSCI.15-03-02354.1995 PMC6578138

[16] McLaughlin M, Hale R, Ellston D, Gaudet S, Lue RA, et al (2002) The distribution and function of alternatively spliced insertions in hD1g. J Biol Chem 277: 6406–6412. 11723125 10.1074/jbc.M108724200

[17] Nguyen MM, Rivera C, Griep AE (2005) Localization of PDZ domain containing proteins Discs Large-1 and Scribble in the mouse eye. Mol Vis 11: 1183–1199. 16402019

[18] Thomas U, Phannavong B, Muller B, Garner CC, Gundelfinger ED (1997) Functional Expression of Rat Synapse-Associated Proteins Sap97 and Sap102 in Drosophila Dlg-1 Mutants - Effects On Tumor Suppression and Synaptic Bouton Structure. Mech Dev 62: 161–174. 9152008 10.1016/s0925-4773(97)00658-8

[19] Rafael JA, Hutchinson TL, Lumeng CN, Marfatia SM, Chishti AH, et al (1998) Localization of Dlg at the mammalian neuromuscular junction. Neuroreport 9: 2121–2125. 9674605 10.1097/00001756-199806220-00039

[20] Laprise P, Viel A, Rivard N (2004) Human homolog of disc-large is required for adherens junction assembly and differentiation of human intestinal epithelial cells. J Biol Chem 279: 10157–10166. 14699157 10.1074/jbc.M309843200

[21] Dow LE, Brumby AM, Muratore R, Coombe ML, Sedelies KA, et al (2003) hScrib is a functional homologue of the Drosophila tumour suppressor Scribble. Oncogene 22: 9225–9230. 14681682 10.1038/sj.onc.1207154

[22] Ishidate T, Matsumine A, Toyoshima K, Akiyama T (2000) The APC-hDLG complex negatively regulates cell cycle progression from the G0/G1 to S phase. Oncogene 19: 365–372. 10656683 10.1038/sj.onc.1203309

[23] Valiente M, Andres-Pons A, Gomar B, Torres J, Gil A, et al (2005) Binding of PTEN to specific PDZ domains contributes to PTEN protein stability and phosphorylation by microtubule-associated serine/threonine kinases. J Biol Chem 280: 28936–28943. 15951562 10.1074/jbc.M504761200

[24] Kim E, Niethammer M, Rothschild A, Jan YN, Sheng M (1995) Clustering of Shaker-type K+ channels by interaction with a family of membrane-associated guanylate kinases. Nature 378: 85–88. 7477295 10.1038/378085a0

[25] Yamamoto Y, Irie K, Asada M, Mino A, Mandai K, et al (2004) Direct binding of the human homologue of the Drosophila disc large tumor suppressor gene to seven-pass transmembrane proteins, tumor endothelial marker 5 (TEM5), and a novel TEM5-like protein. Oncogene 23: 3889–3897. 15021905 10.1038/sj.onc.1207495

[26] Lee S, Fan S, Makarova O, Straight S, Margolis B (2002) A novel and conserved protein-protein interaction domain of mammalian Lin-2/CASK binds and recruits SAP97 to the lateral surface of epithelia. Mol Cell Biol 22: 1778–1791. 11865057 10.1128/MCB.22.6.1778-1791.2002 PMC135599

[27] Kiyono T, Hiraiwa A, Fujita M, Hayashi Y, Akiyama T, et al (1997) Binding of high-risk human papillomavirus E6 oncoproteins to the human homologue of the Drosophila discs large tumor suppressor protein. Proc Natl Acad Sci U S A 94: 11612–11616. 9326658 10.1073/pnas.94.21.11612 PMC23554

[28] Lee SS, Weiss RS, Javier RT (1997) Binding of human virus oncoproteins to hDlg/SAP97, a mammalian homolog of the Drosophila discs large tumor suppressor protein. Proc Natl Acad Sci U S A 94: 6670–6675. 9192623 10.1073/pnas.94.13.6670 PMC21216

[29] Suzuki T, Ohsugi Y, Uchida-Toita M, Akiyama T, Yoshida M (1999) Tax oncoprotein of HTLV-1 binds to the human homologue of Drosophila discs large tumor suppressor protein, hDLG, and perturbs its function in cell growth control. Oncogene 18: 5967–5972. 10557085 10.1038/sj.onc.1203008

[30] Shai A, Brake T, Somoza C, Lambert PF (2007) The human papillomavirus E6 oncogene dysregulates the cell cycle and contributes to cervical carcinogenesis through two independent activities. Cancer Res 67: 1626–1635. 17308103 10.1158/0008-5472.CAN-06-3344 PMC2859178

[31] Frese KK, Latorre IJ, Chung SH, Caruana G, Bernstein A, et al (2006) Oncogenic function for the Dlg1 mammalian homolog of the Drosophila discs-large tumor suppressor. Embo J 25: 1406–1417. 16511562 10.1038/sj.emboj.7601030 PMC1422156

[32] Simonson SJ, Difilippantonio MJ, Lambert PF (2005) Two distinct activities contribute to human papillomavirus 16 E6’s oncogenic potential. Cancer Res 65: 8266–8273. 16166303 10.1158/0008-5472.CAN-05-1651

[33] Hirata A, Higuchi M, Niinuma A, Ohashi M, Fukushi M, et al (2004) PDZ domain-binding motif of human T-cell leukemia virus type 1 Tax oncoprotein augments the transforming activity in a rat fibroblast cell line. Virology 318: 327–336. 14972558 10.1016/j.virol.2003.10.006

[34] Caruana G, Bernstein A (2001) Craniofacial dysmorphogenesis including cleft palate in mice with an insertional mutation in the discs large gene. Mol Cell Biol 21: 1475–1483. 11238884 10.1128/MCB.21.5.1475-1483.2001 PMC86693

[35] Naim E, Bernstein A, Bertram JF, Caruana G (2005) Mutagenesis of the epithelial polarity gene, discs large 1, perturbs nephrogenesis in the developing mouse kidney. Kidney Int 68: 955–965. 16105026 10.1111/j.1523-1755.2005.00489.x

[36] Nguyen MM, Nguyen ML, Caruana G, Bernstein A, Lambert PF, et al (2003) Requirement of PDZ-containing proteins for cell cycle regulation and differentiation in the mouse lens epithelium. Mol Cell Biol 23: 8970–8981. 14645510 10.1128/MCB.23.24.8970-8981.2003 PMC309609

[37] Feng W, Long JF, Fan JS, Suetake T, Zhang M (2004) The tetrameric L27 domain complex as an organization platform for supramolecular assemblies. Nat Struct Mol Biol 11: 475–480. 15048107 10.1038/nsmb751

[38] Mahoney ZX, Sammut B, Xavier RJ, Cunningham J, Go G, et al (2006) Discs-large homolog 1 regulates smooth muscle orientation in the mouse ureter. Proc Natl Acad Sci U S A 103: 19872–19877. 17172448 10.1073/pnas.0609326103 PMC1750896

[39] Iizuka-Kogo A, Ishidao T, Akiyama T, Senda T (2007) Abnormal development of urogenital organs in Dlgh1-deficient mice. Development 134: 1799–1807. 17435047 10.1242/dev.02830

[40] Rivera C, Yamben IF, Shatadal S, Waldof M, Robinson ML, et al (2009) Cell-autonomous requirements for Dlg-1 for lens epithelial cell structure and fiber cell morphogensis. Dev Dyn 238: 2292–2308. 19623611 10.1002/dvdy.22036 PMC3016059

[41] Lakso M, Pichel JG, Gorman JR, Sauer B, Okamoto Y, et al (1996) Efficient in vivo manipulation of mouse genomic sequences at the zygote stage. Proc Natl Acad Sci U S A 93: 5860–5865. 8650183 10.1073/pnas.93.12.5860 PMC39152

[42] Murdoch JN, Henderson DJ, Doudney K, Gaston-Massuet C, Phillips HM, et al (2003) Disruption of scribble (Scrb1) causes severe neural tube defects in the circletail mouse. Hum Mol Genet 12: 87–98. 12499390 10.1093/hmg/ddg014

[43] Rachel RA, Murdoch JN, Beermann F, Copp AJ, Mason CA (2000) Retinal axon misrouting at the optic chiasm in mice with neural tube closure defects. Genesis 27: 32–47. 10862153 10.1002/1526-968x(200005)27:1<32::aid-gene50>3.0.co;2-t

[44] Murdoch JN, Doudney K, Paternotte C, Copp AJ, Stanier P (2001) Severe neural tube defects in the loop-tail mouse result from mutation of Lpp1, a novel gene involved in floor plate specification. Hum Mol Genet 10: 2593–2601. 11709546 10.1093/hmg/10.22.2593

[45] Torban E, Wang HJ, Patenaude AM, Riccomagno M, Daniels E, et al (2007) Tissue, cellular and sub-cellular localization of the Vangl2 protein during embryonic development: effect of the Lp mutation. Gene Expr Patterns 7: 346–354. 16962386 10.1016/j.modgep.2006.07.007

[46] Murdoch JN, Rachel RA, Shah S, Beermann F, Stanier P, et al (2001) Circletail, a new mouse mutant with severe neural tube defects: chromosomal localization and interaction with the loop-tail mutation. Genomics 78: 55–63. 11707073 10.1006/geno.2001.6638

[47] Keller R (2002) Shaping the vertebrate body plan by polarized embryonic cell movements. Science 298: 1950–1954. 12471247 10.1126/science.1079478

[48] Dabdoub A, Donohue MJ, Brennan A, Wolf V, Montcouquiol M, et al (2003) Wnt signaling mediates reorientation of outer hair cell stereociliary bundles in the mammalian cochlea. Development 130: 2375–2384. 12702652 10.1242/dev.00448

[49] Hartmann C (2006) A Wnt canon orchestrating osteoblastogenesis. Trends Cell Biol 16: 151–158. 16466918 10.1016/j.tcb.2006.01.001

[50] Qian D, Jones C, Rzadzinska A, Mark S, Zhang X, et al (2007) Wnt5a functions in planar cell polarity regulation in mice. Dev Biol 306: 121–133. 17433286 10.1016/j.ydbio.2007.03.011 PMC1978180

[51] Kibar Z, Vogan KJ, Groulx N, Justice MJ, Underhill DA, et al (2001) Ltap, a mammalian homolog of Drosophila Strabismus/Van Gogh, is altered in the mouse neural tube mutant Loop-tail. Nat Genet 28: 251–255. 11431695 10.1038/90081

[52] Wang J, Hamblet NS, Mark S, Dickinson ME, Brinkman BC, et al (2006) Dishevelled genes mediate a conserved mammalian PCP pathway to regulate convergent extension during neurulation. Development 133: 1767–1778. 16571627 10.1242/dev.02347 PMC4158842

[53] Wang Y, Guo N, Nathans J (2006) The role of Frizzled3 and Frizzled6 in neural tube closure and in the planar polarity of inner-ear sensory hair cells. J Neurosci 26: 2147–2156. 16495441 10.1523/JNEUROSCI.4698-05.2005 PMC6674805

[54] Curtin JA, Quint E, Tsipouri V, Arkell RM, Cattanach B, et al (2003) Mutation of Celsr1 disrupts planar polarity of inner ear hair cells and causes severe neural tube defects in the mouse. Curr Biol 13: 1129–1133. 12842012 10.1016/s0960-9822(03)00374-9

[55] Lu X, Borchers AG, Jolicoeur C, Rayburn H, Baker JC, et al (2004) PTK7/CCK-4 is a novel regulator of planar cell polarity in vertebrates. Nature 430: 93–98. 15229603 10.1038/nature02677

[56] Montcouquiol M, Sans N, Huss D, Kach J, Dickman JD, et al (2006) Asymmetric localization of Vangl2 and Fz3 indicate novel mechanisms for planar cell polarity in mammals. J Neurosci 26: 5265–5275. 16687519 10.1523/JNEUROSCI.4680-05.2006 PMC6674235

[57] Yao R, Natsume Y, Noda T (2004) MAGI-3 is involved in the regulation of the JNK signaling pathway as a scaffold protein for frizzled and Ltap. Oncogene 23: 6023–6030. 15195140 10.1038/sj.onc.1207817

[58] Hering H, Sheng M (2002) Direct interaction of Frizzled-1, -2, -4, and -7 with PDZ domains of PSD-95. FEBS Lett 521: 185–189. 12067714 10.1016/s0014-5793(02)02831-4

[59] Wawrzak D, Luyten A, Lambaerts K, Zimmermann P (2009) Frizzled-PDZ scaffold interactions in the control of Wnt signaling. Adv Enzyme Regul 49: 98–106. 19534027 10.1016/j.advenzreg.2009.01.002

[60] Van Campenhout CA, Eitelhuber A, Gloeckner CJ, Giallonardo P, Gegg M, et al (2011) Dlg3 trafficking and apical tight junction formation is regulated by Nedd4 and Nedd4–2 E3 ubiquitin ligases. Dev Cell 21: 479–491. 21920314 10.1016/j.devcel.2011.08.003 PMC4452538

[61] Bastock R, Strutt H, Strutt D (2003) Strabismus is asymmetrically localised and binds to Prickle and Dishevelled during Drosophila planar polarity patterning. Development 130: 3007–3014. 12756182 10.1242/dev.00526

[62] Park M, Moon RT (2002) The planar cell-polarity gene stbm regulates cell behaviour and cell fate in vertebrate embryos. Nat Cell Biol 4: 20–25. 11780127 10.1038/ncb716

[63] Topczewski J, Dale RM, Sisson BE (2011) Planar cell polarity signaling in craniofacial development. Organogenesis 7: 255–259. 22134372 10.4161/org.7.4.18797 PMC3265827

[64] Bekman E, Henrique D (2002) Embryonic expression of three mouse genes with homology to the Drosophila melanogaster prickle gene. Mech Dev 119 Suppl 1S77–81. 14516664 10.1016/s0925-4773(03)00095-9

[65] Darken RS, Scola AM, Rakeman AS, Das G, Mlodzik M, et al (2002) The planar polarity gene strabismus regulates convergent extension movements in Xenopus. EMBO J 21: 976–985. 11867525 10.1093/emboj/21.5.976 PMC125882

[66] De Calisto J, Araya C, Marchant L, Riaz CF, Mayor R (2005) Essential role of non-canonical Wnt signalling in neural crest migration. Development 132: 2587–2597. 15857909 10.1242/dev.01857

[67] Yamaguchi TP, Bradley A, McMahon AP, Jones S (1999) A Wnt5a pathway underlies outgrowth of multiple structures in the vertebrate embryo. Development 126: 1211–1223. 10021340 10.1242/dev.126.6.1211

[68] He F, Xiong W, Yu X, Espinoza-Lewis R, Liu C, et al (2008) Wnt5a regulates directional cell migration and cell proliferation via Ror2-mediated noncanonical pathway in mammalian palate development. Development 135: 3871–3879. 18948417 10.1242/dev.025767 PMC3010758

[69] Barrow J (2011) Wnt/planar cell polarity signaling: an important mechanism to coordinate growth and patterning in the limb. Organogenesis 7: 260–266. 22198433 10.4161/org.7.4.19049 PMC3265828

[70] Romereim SM, Dudley AT (2011) Cell polarity: The missing link in skeletal morphogenesis? Organogenesis 7: 217–228. 22064549 10.4161/org.7.3.18583 PMC3243035

[71] Wyngaarden LA, Vogeli KM, Ciruna BG, Wells M, Hadjantonakis AK, et al (2010) Oriented cell motility and division underlie early limb bud morphogenesis. Development 137: 2551–2558. 20554720 10.1242/dev.046987 PMC2927701

[72] Gros J, Hu JK, Vinegoni C, Feruglio PF, Weissleder R, et al (2010) WNT5A/JNK and FGF/MAPK pathways regulate the cellular events shaping the vertebrate limb bud. Curr Biol 20: 1993–2002. 21055947 10.1016/j.cub.2010.09.063 PMC2998074

[73] Wang B, Sinha T, Jiao K, Serra R, Wang J (2011) Disruption of PCP signaling causes limb morphogenesis and skeletal defects and may underlie Robinow syndrome and brachydactyly type B. Hum Mol Genet. 20: 271–285. 10.1093/hmg/ddq462 PMC3031336 20962035

[74] Gao B, Song H, Bishop K, Elliot G, Garrett L, et al (2011) Wnt signaling gradients establish planar cell polarity by inducing Vangl2 phosphorylation through Ror2. Dev Cell 20: 163–176. 21316585 10.1016/j.devcel.2011.01.001 PMC3062198

[75] Sasaki T, Ito Y, Bringas P Jr, Chou S, Urata MM, et al (2006) TGFbeta-mediated FGF signaling is crucial for regulating cranial neural crest cell proliferation during frontal bone development. Development 133: 371–381. 16368934 10.1242/dev.02200

[76] Trokovic N, Trokovic R, Mai P, Partanen J (2003) Fgfr1 regulates patterning of the pharyngeal region. Genes Dev 17: 141–153. 12514106 10.1101/gad.250703 PMC195961

[77] Ito Y, Yeo JY, Chytil A, Han J, Bringas P Jr, et al (2003) Conditional inactivation of Tgfbr2 in cranial neural crest causes cleft palate and calvaria defects. Development 130: 5269–5280. 12975342 10.1242/dev.00708

[78] Dudas M, Sridurongrit S, Nagy A, Okazaki K, Kaartinen V (2004) Craniofacial defects in mice lacking BMP type I receptor Alk2 in neural crest cells. Mech Dev 121: 173–182. 15037318 10.1016/j.mod.2003.12.003

[79] Brault V, Moore R, Kutsch S, Ishibashi M, Rowitch DH, et al (2001) Inactivation of the beta-catenin gene by Wnt1-Cre-mediated deletion results in dramatic brain malformation and failure of craniofacial development. Development 128: 1253–1264. 11262227 10.1242/dev.128.8.1253

[80] Day TF, Guo X, Garrett-Beal L, Yang Y (2005) Wnt/beta-catenin signaling in mesenchymal progenitors controls osteoblast and chondrocyte differentiation during vertebrate skeletogenesis. Dev Cell 8: 739–750. 15866164 10.1016/j.devcel.2005.03.016

[81] Hill TP, Spater D, Taketo MM, Birchmeier W, Hartmann C (2005) Canonical Wnt/beta-catenin signaling prevents osteoblasts from differentiating into chondrocytes. Dev Cell 8: 727–738. 15866163 10.1016/j.devcel.2005.02.013

[82] Joeng KS, Schumacher CA, Zylstra-Diegel CR, Long F, Williams BO (2011) Lrp5 and Lrp6 redundantly control skeletal development in the mouse embryo. Dev Biol 359: 222–229. 21924256 10.1016/j.ydbio.2011.08.020 PMC3220949

